# Life Satisfaction Before and During COVID-19 Pandemic in Thailand

**DOI:** 10.3389/ijph.2023.1605483

**Published:** 2023-07-13

**Authors:** Sirinya Phulkerd, Sasinee Thapsuwan, Rossarin Soottipong Gray, Aphichat Chamratrithirong, Umaporn Pattaravanich, Chantana Ungchusak, Pairoj Saonuam

**Affiliations:** ^1^ Institute for Population and Social Research, Mahidol University, Nakhon Pathom, Thailand; ^2^ Healthy Lifestyle Promotion Section of Thai Health Promotion Foundation, Bangkok, Thailand

**Keywords:** COVID-19, sociodemographic factors, healthy lifestyle, life satisfaction, socioeconomic factors, Thailand

## Abstract

**Objective:** To investigate prevalence of life satisfaction in the Thai population before and during the COVID-19 epidemic, and factors associated with life satisfaction during the epidemic.

**Methods:** Multistage sampling was used to draw a sample from the Thai population. A total of 3,115 Thai participants age 15 years or older from a nationally-representative longitudinal survey in 2019 and in 2021 were included in this study. The study applied the Scale with Life Satisfaction (SWLS) instrument to measure life satisfaction among the Thai population before and during the COVID-19 epidemic. Multiple regression analysis was used to investigate the association between life satisfaction and other variables. The follow-up survey response rate for individuals was 44.8%.

**Results:** An average life satisfaction score during the COVID-19 epidemic (in 2021) was 22.4 which decreased from 25.5 before the COVID-19 epidemic (in 2019). More than one-third of the participants (36.5%) reported having less life satisfaction during the epidemic, which was nearly 20 percentage points higher than before the epidemic (17.7%). Controlling for life satisfaction in 2019, the analysis found statistical associations between demographic and economic characteristics and health-related behaviours, and life satisfaction during 2021. People in the older age cohorts (*p* ≤ 0.001), in a rural area (*p* ≤ 0.05), having higher education (*p* ≤ 0.001), still being employed (*p* ≤ 0.01) and becoming unemployed (*p* ≤ 0.01) had higher life satisfaction. The possibility of higher life satisfaction was also found in people who maintained good health (*p* ≤ 0.01), sufficient physical activity (*p* ≤ 0.001), and fruit and vegetable intake (*p* ≤ 0.01). People with income loss during the epidemic had lower life satisfaction (*p* ≤ 0.05).

**Conclusion:** The findings suggest that policies and systems for resilience and social protection are needed for empowering individuals—especially the poor and vulnerable—to cope with crises, and improve health and wellbeing outcomes.

## Introduction

The COVID-19 pandemic has had both positive and negative psychological effects on populations worldwide. Recent COVID-19 studies have demonstrated such positive benefits for people as more opportunity for strengthening family relationships and finding new hobbies [1], and for reducing air pollution, improving air and water quality and lowering noise levels [2]. However, more studies have documented the adverse effects of COVID-19 and harsh control measures on psychological outcomes, such as anxiety, distress, depression, anger, and fear [3] that can erode wellbeing and life satisfaction [4, 5]. This unhealthy state may impact negatively on physical health and longevity [6]. It is also well-known that wellbeing in childhood is predictive of wellbeing in adulthood [7].

The spread of COVID-19 has an impact on individual life satisfaction in many countries. Previous studies showed the negative correlation between life satisfaction and COVID-19 in Northern Europe; however, there was insignificant correlation in Southern and Western Europe [8]. A study with university students in European and Latin America countries found that poor self-rated physical health was a distinct predictor of low life satisfaction during the pandemic [9]. Another study in Poland investigated an influence of fear of COVID-19 on life satisfaction during the pandemic [10]. The results showed a negative association between these two variables that health-related hardiness and sense of coherence were found to be important mediators between them. These findings point to the importance of giving special public health attention that should be focused on psychologically supporting people during and after the pandemic.

The COVID-19 epidemic in Thailand began in March 2020 (as the 1st wave), where the first outbreaks were traced to a group of spectators at a boxing event and a cluster of nighttime entertainment establishments. COVID-19 then spread to 68 of Thailand’s 77 provinces [11]. Harsh government containment measures (e.g., closing international borders, closing nighttime entertainment establishments, closing schools, nighttime curfews, area-specific lockdowns, etc.) managed to slow spread of COVID-19 to a trickle for most of the rest of 2020. However, as the virus evolved into more infectious variants, new outbreaks occurred, starting with a 2nd wave in December 2020, a 3rd wave in April 2021, a 4th wave in June 2021, and a 5th wave in January 2022. Research studies started to report the negative effects of the epidemic on mental health of Thais. University students reported having stress, anxiety, and depression during the epidemic [12]. The anxiety was the most common affliction among students, followed by depression, and stress. One study examined the effect of the epidemic on wellness of students enrolled in health profession curricula and found elevated levels of anxiety [13]. That study also reported increased sedentary behavior and undesirable weight gain. Thai healthcare workers also experienced negative mental health outcomes during the epidemic, such as burnout, anxiety, depression, and post-traumatic stress [14].

Despite a growing number of studies on the impact of the COVID-19 epidemic on various aspects of mental health outcomes among Thais, there is limited evidence on how the epidemic may be affecting general wellbeing of the population, especially life satisfaction. The United Nations Population Fund reported that Thai older persons had lower life satisfaction during the epidemic compared with before COVID-19 [15]. Those in urban areas were twice as likely as their rural counterparts to report lower life satisfaction. Another study found a positive correlation between life satisfaction and receiving government relief payments and other financial support for the working-age population [16]. As yet, no studies have investigated the prevalence of life satisfaction among Thais at the population level and across age groups, with a comparison before and during the epidemic. In addition, there has not been a clear identification of determinants of vulnerability to life satisfaction during a national disaster such as COVID-19.

Therefore, this study investigated the prevalence of life satisfaction in the Thai population before and during the COVID-19 epidemic, and identified factors associated with individual life satisfaction during the epidemic. Findings of this study should help the Thai government and other stakeholders to better understand the situation of life satisfaction among Thais across age groups. This information can be used to design programs and interventions to restore life satisfaction to pre-epidemic levels, and intensify the effort to “leave no one behind” which is the central promise of the 2030 Sustainable Development Agenda. Ultimately, it can be expected that improved wellbeing, especially among vulnerable groups, will contribute to better individual performance at work [17], as well as better national economic performance for the nation as a whole [18].

## Methods

### Study Design and Participants

This study used individual-level data from a nationally-representative, population-based longitudinal survey of the Thai population in 2019 (pre-epidemic) and in 2021 (during the 3rd and 4th waves) that follows food consumption, health behaviour, and wellbeing of Thais age 6 years or older.

This study focused on the sub-group of the sample age 15 years or older who responded to questions on life satisfaction. The study recruited participants in the 2019 survey using multistage stratified sampling design to obtain a representative sample of Thai population that can generate generalisable findings of the study. The sampling design and sample size calculation were conducted by the National Statistical Office of Thailand (NSO) - the Thai government’s official statistics surveyor - which is responsible for conducting and facilitating census and sample surveys for national statistical information database.

In the 2019 survey the NSO conducted the stratified sampling in four stages. The first stage involved a systematic sampling of two provinces within each geographic region. The selected provinces were Nakhon Pathom and Prachin Buri in Central region, Nakhon Sawan and Lampang in Northern region, Surin and Udon Thani in Northeast region, and Songkhla and Phatthalung in South region. Bangkok was also included as it is the sole special administrative area in Thailand. In total, nine provinces were sampled in this study. In the second stage, a systematic sampling of postal districts within each province by considering administrative boundaries (urban and rural areas) was conducted. This involved the selection of enumeration areas (EAs) and households (20 households in each EA). A household list was provided by the National Statistical Office. In the third stage, a systematic sampling of households nested within each selected EA was obtained. Each household was then contacted for study participation. At the final stage, all the household members who met the following inclusion criteria were invited to take part in the study.

Inclusion criteria for participation in this study were that a participant must have/have been: 1) lived in the selected household, 2) available during a visit by research team, 3) at least 15 years of age, 4) fluent in Thai (i.e., able to speak, read and write), and willing and able to participate in a face-to-face interview. A participant who had a condition or was in a situation which may have put him/her at significant risk, or may have confounded the study results was excluded.

The 2021 survey was a nested study within the 2019 survey. All the 2019 participants were recruited to take part the 2021 survey.

### Data Collection

Of the total 3,720 sampled households in the initial year, 3,297 households in 2019 (response rate 88.6%) and 2,647 households in 2021 (follow-up rate 80.3%) successfully participated in the survey (Table 1). Participant recruitment was conducted during 1st July in 2019 and 15th December in 2021. The total number of respondents who successfully completed the survey in 2019 and both surveys in 2019 and 2021 was 6,956 and 3,115 (follow-up rate 44.8%), respectively. Each respondent was interviewed in person by the research team. Responses of the respondents were recorded in a tablet computer, and then uploaded to Qualtrics when an Internet connection was available.

**TABLE 1:** Number of sampled households and respondents in 2019 and 2021 (Thailand, 2019 and 2021).

Region	Sampled households	Number of households and respondents which completed the survey			
		2019		2021	
		Households	Respondents	Households	Respondents (who also participated in 2019 survey)
Bangkok	620	513	741	506	652
Central	660	584	1,275	469	581
North	720	652	1,284	530	466
Northeast	920	814	2,044	687	796
South	800	734	1,613	455	620
Total	3,720	3,297	6,956	2,647	3,115

### Measurement

The study questionnaires used in 2019 and 2021 include questions on demographic and economic characteristics, life satisfaction and health-related factors (available in Supplementary Information).

### Study Variables

#### Life Satisfaction

This study used life satisfaction score as the outcome variable to measure life satisfaction among the Thai population before and during the COVID-19 epidemic. The study applied the Scale with Life Satisfaction (SWLS) instrument, developed by Diener and colleagues [19] which codes life satisfaction across a 7-level scale. The SWLS has been proven as a valid and reliable tool, and it is widely used in many countries and in diverse populations. This study used the Thai-language version of the SWLS which is available at https://eddiener.com/scales/7. The tool was assessed for an internal consistency and reliability using Cronbach’s alpha prior to data collection. The alpha value of the Thai SWLS is reported elsewhere [20].

Life satisfaction was measured by response to following statements: 1) In most ways my life is close to ideal; 2) The conditions of my life are excellent; 3) I am satisfied with my life; 4) So far, I have gotten the important things I want out of life; and 5) If I could live my life over, I would change almost nothing.

The individual respondent was asked to specify their level of agreement with each statement on a seven-point scale: 1) Strongly disagree; 2) Disagree; 3) Slightly disagree; 4) Neither agree nor disagree; 5) Slightly agree; 6) Agree; and 7) Strongly agree. Each statement was assigned one to seven points. The respondent’s score is the sum of the level of agreement with the five statements, generating a potential range from 5 to 35 points. A higher score indicates a higher level of life satisfaction.

#### Demographic and Economic Characteristics

This study used demographic and economic characteristics as independent variables. Demographic and economic variables are sex, age, marital status, place of residence, education, employment status, and income. The study used the latest demographic and economic data on sex, age, place of residence, and education in 2021, and the data from 2019 to 2021 for changes in marital status, place of residence, and job status. This is because previous literature reported significant changes in marital status, employment status, and income during the COVID-19 epidemic [21–23]. Details of each variable in the analysis are described below.

Sex was coded as male or female.

Age was coded according to age classification of the system of the National Statistical Office (NSO). The study classified age into four groups: Early-working age (15–29 years); middle-working age (30–44 years); late-working age (45–59 years); and older person (60 years or older).

Place of residence was coded as rural or urban.

Educational attainment was coded as completing primary school education or lower; completing secondary school education or equivalent; and completing a Bachelor’s degree or higher.

Marital status was coded for the respondent’s status before and during COVID-19 as follows: single/single; single/married; married/married; married/widowed; divorced, separated, widowed, divorced, or separated/widowed; and divorced or separated, widowed, divorced or separated/married.

Employment status was coded for a respondent’s status in 2019 and 2021 as follows: unemployed/unemployed, employed/employed, employed/unemployed, and unemployed/employed.

Income was coded for change in the respondent’s monthly income before and during COVID-19: more income; less income; and same income.

#### Health-Related Factors

Health status, physical activity and fruit and vegetable consumption were included in the analysis.

For the health status variable, each respondent was asked to self-assess his or her personal health status. For example, the respondent was asked if they currently had a chronic disease(s), and response was coded either “yes” or “no.” Health status was coded for the period before and during the COVID-19 epidemic as follows: yes/yes, no/yes, yes/no, and no/no.

For physical activity variable, respondents were assessed whether they achieved a sufficient level of physical activity per day with the following question: “Do you engage in physical activity for at least 30 min per day and at least 3 days per week (i.e., totalling 210 min a week of moderate intensity activity, e.g., brisk walking, running, aerobics, competitive games, and sports)?” Response was coded “yes” or “no.” Physical activity was coded for the period before and during the COVID-19 as follows: insufficient/insufficient, sufficient/insufficient, insufficient/sufficient and sufficient/sufficient.

For fruit and vegetable consumption, respondents were asked the following questions:

1. “Did you eat the following fruits/vegetables in the past week?”
2. ‘If yes, how many days did you eat them per week?”
3. “How many times a day did you eat them?”
4. “How much is the average daily amount (in rice ladle unit) of your consumption of each fruit/vegetable group?”

After completing the last question, the average daily amount reported was converted into number of grams based on the standard serving adapted from the Thailand Nutrition Flag [24]. Responses from Questions #2–4 were then calculated for average consumption per person (grams per day) of fruits and vegetables combined, by multiplying all answers from these questions and then dividing by seven.

Accordingly, each respondent was categorised into two groups: “Sufficient consumption (eating at least 400 g of fruits/vegetables combined per day [25])” and “Insufficient consumption (eating less than 400 g of fruits/vegetables combined per day).”

The fruit and vegetable consumption variable was coded for the data reported before and during the COVID-19 epidemic as follows: insufficient/insufficient, sufficient/insufficient, insufficient/sufficient and sufficient/sufficient.

### Statistical Analysis

This study analysed the population-representative survey data in 2019 and 2021 to determine individual levels of life satisfaction, and associations between life satisfaction and the independent variables. The study measured the levels of life satisfaction using descriptive statistics (frequency and percentage), and the relationship between independent variables and life satisfaction in 2021 using ANOVA. The multiple regression analysis was also employed to analyse the association between life satisfaction during the COVID-19 and each of the independent variables, adjusting for the covariates sequentially, controlling for life satisfaction before the COVID-19.

The dependent variable of the analysis was life satisfaction. The independent variables in the multiple regression equation were age, gender, marital status, place of residence, education, employment, income, health status, physical activity and fruit & vegetable consumption. Any relationship with a *p*-value of 0.05 or less (2-tailed) was considered statistically significant. All analyses were performed with SPSS Statistics Version 18.

## Results

### Life Satisfaction Levels

An average life satisfaction score during the COVID-19 epidemic (in 2021) was 22.4 which decreased from 25.5 before the COVID-19 epidemic (in 2019) (Table 2). Life satisfaction scores were divided into two groups: 1) Less life satisfaction (scores 5–20); and 2) More life satisfaction (scores 21–35) [26]. Of the total 3,115 respondents, 36.5% reported having less life satisfaction during the epidemic, which was nearly 20 percentage points higher than before the epidemic (17.7%).

**TABLE 2:** Mean and median scores of life satisfaction of the Thai population before and during the COVID-19 epidemic (N = 3,115) (Thailand, 2019 and 2021).

Life satisfaction level	Before COVID-19 (2019)		During COVID-19 (2021)	
	N	%	N	%
Less life satisfaction (scores 5–20)	551	17.7	1,136	36.5
More life satisfaction (scores 21–35)	2,564	82.3	1,979	63.5
Mean (SD)	25.5 (5.115)		22.4 (5.618)	
Median	26.0		23.0	
Total	3,115	100.0	3,115	100.0

Table 3 presents general characteristics of the respondents, and distributions and changes (in brackets) in life satisfaction scores before and during the COVID-19 epidemic. Respondents who were female (−3.2), were age 15–29 years (−3.7), lived in an urban area (−3.4), and attained secondary school education or equivalent (−3.3) had the greatest decrease in life satisfaction compared with other groups. Respondents who had status changes before and during the epidemic in terms of marital status (from being widowed, divorced or separated to married) (−3.7), employment status (from being employed to unemployed) (−3.7), and income status (to less income) (−3.5) had the greatest decrease in life satisfaction.

**TABLE 3:** Distribution and change in mean score of life satisfaction before and during the COVID-19 epidemic (N = 3,115) (Thailand, 2019 and 2021).

Variables	N	%	Life satisfaction score				
			2019	2021	F (2021)	Change	F (change)
Gender					1.111		1.987
Male	1,096	35.2	25.5	22.7		−2.8	
Female	2,019	64.8	25.4	22.2		−3.2	
Age (years)					8.595***		2.926*
15–29	353	11.3	25.5	21.8		−3.7	
30–44	528	17.0	25.4	22.3		−3.1	
45–59	1,066	34.2	25.3	21.8		−3.5	
60 or older	1,168	37.5	25.7	23.1		−2.6	
Marital status (2019/2021)					2.443*		0.844
Single/Single	404	13.0	25.5	22.3		−3.2	
Single/Married	25	0.8	24.9	23.7		−1.2	
Married/Married	2,014	64.6	25.7	22.6		−3.1	
Married/Widowed, divorced or separated	127	4.1	24.9	21.2		−3.7	
Widowed, divorced or separated/Widowed, divorced or separated	505	16.2	24.8	21.9		−2.9	
Widowed, divorced or separated/Married	40	1.3	26.6	22.9		−3.7	
Place of residence					5.337*		11.330**
Urban	1,860	59.7	25.7	22.3		−3.4	
Rural	1,255	40.3	25.1	22.5		−2.6	
Education					12.886***		0.595
Primary school or lower	1,823	58.5	25.2	22.1		−3.1	
Secondary school or equivalent	1,019	32.7	25.6	22.3		−3.3	
Bachelor’s degree or higher	273	8.8	26.6	24.2		−2.4	
Employment status (2019/2021)					5.095**		3.928**
Unemployed/Unemployed	890	28.6	25.4	22.9		−2.5	
Employed/Employed	1,609	51.7	25.6	22.3		−3.3	
Employed/Unemployed	403	12.9	25.3	21.6		−3.7	
Unemployed/Employed	213	6.8	24.9	22.2		−2.7	
Income status (2019/2021)					1.055		4.133*
Same income	1,674	53.7	25.3	22.4		−2.9	
Less income	1,175	37.7	25.7	22.2		−3.5	
More income	266	8.6	25.2	22.7		−2.5	
Health status—presence of chronic disease(s) (2019/2021)					12.468***		10.311***
Yes/Yes	154	4.9	22.4	20.8		−1.6	
No/Yes	332	10.7	24.7	21.2		−3.5	
Yes/No	210	6.7	22.9	21.8		−1.1	
No/No	2,419	77.7	26.0	22.7		−3.3	
Physical activity (2019/2021)					39.010***		9.612***
Insufficient/Insufficient	1,515	48.6	24.9	21.5		−3.4	
Sufficient/Insufficient	536	17.2	25.9	22.2		−3.7	
Insufficient/Sufficient	494	15.9	25.5	23.1		−2.4	
Sufficient/Sufficient	570	18.3	26.4	24.3		−2.1	
Fruit & vegetable consumption (2019/2021)					11.372***		4.846**
Insufficient/Insufficient	1,371	44.0	25.4	22.2		−3.2	
Sufficient/Insufficient	579	18.6	25.4	21.6		−3.8	
Insufficient/Sufficient	551	17.7	25.4	22.5		−2.9	
Sufficient/Sufficient	614	19.7	25.8	23.4		−2.4	
Total	3,115	100.0	25.5	22.4	-	−3.1	-

*p ≤ 0.05, **p ≤ 0.01, ***p ≤ 0.001.

Some changes in life satisfaction scores were observed in health–related variables. Greatest changes were reported by respondents living with no chronic disease before the epidemic to living with a chronic disease during the epidemic (−3.5), from having sufficient physical activity before the epidemic to having insufficient physical activity during the epidemic (−3.7), and from having sufficient fruit and vegetable consumption before the epidemic to having insufficient fruit and vegetable consumption during the epidemic (−3.8).

### Multiple Regression of Life Satisfaction by Sociodemographic and Socioeconomic Characteristics and Health-Related Factors

Table 4 presents results of the regression analysis of associations between life satisfaction during the COVID-19 and demographic and economic characteristics and health-related factors, controlled by life satisfaction in 2019, before the Thai COVID-19 epidemic.

**TABLE 4:** Multiple regression of life satisfaction by demographic and economic characteristics and health-related factors (N = 3,115) (Thailand, 2019 and 2021).

Variables	Regression			
	B	Std. Error	Β	*p*-value
Life satisfaction score (2019)	0.229	0.019	0.209	***
Sex (References group = Female)				
Male	0.078	0.210	0.007
Age (years) (References group = 15–29)				
30–44	1.079	0.457	0.069	*
45–59	1.148	0.448	0.096	*
60 years or older	2.415	0.458	0.212	***
Marital status (2019/2021) (References group = Single/Single)				
Single/Married	1.791	1.094	0.028	
Married/Married	0.085	0.366	0.007	
Married/Widowed, divorced or separated	−0.929	0.576	−0.033	
Widowed, divorced or separated/Widowed, divorced or separated	−0.467	0.433	−0.031	
Widowed, divorced or separated/Married	0.329	0.908	0.007	
Place of residence (References group = Urban)				
Rural	0.480	0.198	0.042	*
Education (References group = Primary school or lower)				
Secondary school or equivalent	0.449	0.249	0.037	
Bachelor’s degree or higher	1.690	0.366	0.091	***
Employment status (2019/2021) (References group = Unemployed/Unemployed)				
Employed/Employed	−0.919	0.289	−0.082	**
Employed/Unemployed	−1.036	0.338	−0.062	**
Unemployed/Employed	−1.115	0.480	−0.050	*
Income (2019/2021) (References group = Same income)				
Less income	−0.672	0.271	−0.058	*
More income	0.725	0.385	0.036	
Health status—presence of chronic disease(s) (2019/2021) (References group = Yes/Yes)				
No/Yes	−0.087	0.521	−0.005	
Yes/No	0.973	0.564	0.043	
No/No	1.196	0.457	0.089	**
Physical activity (2019/2021) (References group = Insufficient/Insufficient)				
Sufficient/Insufficient	0.475	0.268	0.032	
Insufficient/Sufficient	1.256	0.276	0.082	***
Sufficient/Sufficient	2.125	0.268	0.146	***
Fruit & vegetable consumption (2019/2021) (References group = Insufficient/Insufficient)				
Sufficient/Insufficient	−0.523	0.266	−0.036	
Insufficient/Sufficient	0.278	0.272	0.019	
Sufficient/Sufficient	0.832	0.266	0.059	**
Constant	13.591			
Adjusted R squared	0.118			

*p ≤ 0.05, **p ≤ 0.01, ***p ≤ 0.001.

B—Unstandardized coefficient.

β—Standardized beta coefficient.

Level of life satisfaction in 2021 was strongly associated with level of life satisfaction in 2019 (*β* = 0.209; *p* ≤ 0.001). There were significant associations between life satisfaction and some demographic characteristics, i.e., age, place of residence, and education. Respondents who were at age 60 years or older (*β* = 0.212; *p* ≤ 0.001), resided in a rural area (*β* = 0.042; *p* ≤ 0.05), and had at Bachelor’s degree or higher (*β* = 0.091; *p* ≤ 0.001) were more likely to have higher life satisfaction.

For other economic factors, changes in employment status were found to be significantly associated with life satisfaction. Respondents who were still employed (*β* = −0.082; *p* ≤ 0.01), lost their job (*β* = −0.062; *p* ≤ 0.01), or got hired (*β* = −0.050; *p* ≤ 0.05) during the epidemic were more likely to have lower life satisfaction than those who were jobless during the epidemic. Respondents who earned less income during the epidemic had lower life satisfaction (*β* = −0.058; *p* ≤ 0.05).

Considering other health-related factors, respondents living with no chronic disease before and during the epidemic were more likely to have higher life satisfaction (*β* = 0.089; *p* ≤ 0.01) than other groups. Respondents who had sufficient levels (daily) of physical activity and fruit and vegetable consumption before and during the epidemic were more likely to have higher life satisfaction (*β* = 0.146; *p* ≤ 0.001 and *β* = 0.059; *p* ≤ 0.01, respectively) than other groups.

## Discussion

This study drew upon longitudinal data from a nationally-representative sample of the Thai population to examine contributing factors associated with life satisfaction during the COVID-19 epidemic. This study is the first investigation to compare life satisfaction score and associated factors during the COVID-19 epidemic in Thailand and in the group of lower- and middle-income countries in Asia. The study identified potential predictors of life satisfaction, and suggests that wellbeing is influenced by a number of sociodemographic and health-related factors.

This study highlights a number of key findings from the multiple regression analysis. First, people who reported having life satisfaction before the epidemic remained satisfied with their life during the epidemic. This is not surprising since people with a positive disposition toward life in general may be more resilient in coping with adversity that may arise. In addition, older persons were more likely to have higher life satisfaction than their younger counterparts. This finding is consistent with a previous study in South Korea which found that people age 60 years or older were less dissatisfied with their life than people age 19 and 29 years [27]. This finding can be explained, perhaps, by the older generation’s accumulation of experience and having had more time to develop a lifestyle which helps them cope. Moreover, Thailand has social welfare and health services for older persons, such as long-term care insurance under the Universal Health Coverage scheme, a state pension for retirees from the Social Security Fund, pensions for retired government civil servants, and a National Savings Fund, and a minimal monthly old-age allowance for everyone else which increases with age. These safety nets can help ease the financial burden and improve daily life for senior citizens. This finding also suggests that there is ample opportunity for intergenerational activities that can bring the younger and older generations together to interact, and where older persons can transmit their life experience and lessons learned to the next generation to help them acquire coping skills and strategies to improve life satisfaction.

Other factors affecting life satisfaction include residential area and employment status. People living in an urban area tend to have lower life satisfaction than those living in a rural area. This finding is consistent with previous studies for other countries, in that, people living in cities were more dissatisfied or less satisfied with their life than those living in towns and villages [27–29]. This might be a function of the harsher COVID-19 containment measures and travel restrictions, and mandates for social distancing which are more enforced and harder to comply with in more densely-populated areas of the country. The forced isolation in cities limits social interaction among family, friends, and acquaintances more than it would in a village, all of which erodes life satisfaction.

Changes in employment status also impacted on life satisfaction of Thai population during the COVID-19. A striking finding in the present study is that people who became employed (being jobless before COVID-19) or continued to work during the epidemic had lower life satisfaction compared with people who remained jobless before and during the epidemic. This could be explained by the fact that the invisible, airborne transmission of a lethal pathogen is more threatening to workers in jobs which require them to interact with strangers or many other co-workers. This is particularly true for frontline jobs which are in a physical workplace and in proximity of a constant flow of people who might be carriers of COVID-19. Thus, these types of workers were in high demand during COVID-19, especially healthcare workers, cashiers, personal care workers, food processing workers, construction workers, and assembly workers, among many other types. These workers were, thus, at risk by being employed. Many of these jobs are also concentrated among the young, lower-educated, migrants, ethnic minorities, and other low-skilled workers in minimum-wage jobs. The work itself is stressful even without an epidemic, and the threat of COVID-19 certainly increased the stress and anxiety significantly for these vulnerable members of the labour force [30]. At the same time, the people who lost full-time work during the epidemic had an immediate negative impact on their ability to make ends meet, since many of these workers probably had limited or no savings, and little options for alternative work. Thus, sudden unemployment would certainly erode satisfaction with their life. In particular, the young, lower-educated, migrants, racial/ethnic minorities, and low-skilled workers were at a higher risk of job loss and were expected to recover more slowly than other population groups [31].

This study found an association between income loss and lower life satisfaction, and that is consistent with a previous study [32]. During the COVID-19 epidemic, a wide range of government responses to mitigate and suppress spread of the virus were implemented, which included school closures, travel restrictions, bans on public gatherings, stay-at-home orders, and severe reduction of public transportation [33]. These restrictions unavoidably imposed high social and economic costs on people, especially putting them at higher risk of income loss through working-hour reductions or job termination [30]. Together with differences in household structures and inequalities in access to savings, workers from vulnerable and minority groups and migrants were found to be hit hardest by the containment measures, since they were already living at the margins of the society and economy, and could ill-afford any shocks such as an epidemic [34]. This negative employment change can lead workers in at-risk jobs to suffer particularly large losses in income, and thus erasing any life satisfaction they may have felt before the epidemic.

This study identified other sources of life satisfaction, such as health status and lifestyle behaviours. People who can maintain a healthy lifestyle—living without chronic disease and meeting a sufficient level of physical activity and fruit and vegetable intake reported that the epidemic actually had a positive impact on life satisfaction. The WHO addressed the significant challenge for healthy individuals during stay-at-home restrictions as what they ate, drank, and level of physical activity. Those adaptations are key predictors in a person’s ability to prevent and recover from COVID-19 infection [35, 36]. Unhealthy diet and low levels of physical activity can have negative effects on individual health, mental health, and overall wellbeing. Thus, WHO strongly recommends that individuals remain eating a healthy diet and being physically active during the COVID-related restrictions, as they are essential to avert other health problems, including obesity and noncommunicable diseases or other debilitating conditions. Healthy behaviour maintenance and improvement in Thai wellbeing during the epidemic are possibly the effect of continued implementation of public education campaigns by the government, i.e., to promote a healthy, balanced lifestyle including regular physical activity and nutritious diet to boost the immune system against COVID-19. The findings from this study confirm the importance of resilience among a population during the epidemic through maintaining a heathy lifestyle. More government attention should be given to building resilience at the individual and household levels so that Thais can adapt to difficult or challenging life experiences under uncertain circumstances or sudden adversity they may face.

This study has some limitations. First, data collection used an interviewed questionnaire which relies on an individual’s ability to remember their past experience, i.e., daily levels of physical activity and fruit and vegetable intake, may affect estimates and calculation of such behaviours in the sample. Recall bias could cause an overestimate or underestimate of the various indicators and variables. Measurement of life satisfaction is also reliant on self-reports and is rather subjective it its own right. Thus, the life satisfaction ratings are subject to recall bias and whether a person has an optimistic or pessimistic disposition in general. However, the measurement used in this study has been proved to be valid in various contexts, and has been widely used in examining life satisfaction in various groups of populations and countries with different income levels. That said, this study did not include other variables that may influence life satisfaction, such as exposure to COVID-19, personal and social contact and support, and living conditions [9, 37, 38]. The study also did not analyse differences in life satisfaction and its predictors based on household. Further research should explore the influence of other factors on an individual’s life satisfaction, and conduct a household-level analysis.

Another limitation involves loss to follow-up which is usually a limitation of longitudinal studies. This can lead to attrition bias which can affect the validity and reliability of the study findings. There are significant challenges associated with following participants during the Thai COVID-19 epidemic. For example, fears of the COVID-19, COVID-19 infection and high risk of getting infection can lead to dropping out of the study. Therefore, strategic follow-up plan that can enhance study retention and minimise attrition bias is needed in the future. This can include frequent contact with participants and secondary contacts (i.e., a village leader), creation of connections with relevant local government offices, and complementary to other existing relevant datasets which can strengthen the validity of the study findings.

### Policy Implications

The findings suggest that it is important for the government to address root causes of the demographic and economic disparities, which contribute to persistent structural disadvantages and, thus, negative mental wellbeing faced by groups of the population, but especially vulnerable and marginalised. The government should give priority to building resilience and social protection systems for empowering individuals to cope with crises and shocks, improve productivity, improve health, and improve education outcomes. People need to seek opportunity to lift themselves and their families out of adversity and toward a better life [39]. Well-designed and targeted policies for resilience and social protection can promote self-reliance or self-sufficiency of individuals to meet essential needs or attain an acceptable level of functioning in daily life [40, 41]. Ultimately the goal is to promote greater equity, improve human capital and stimulate economic growth. Social protection has also become a global priority as a key factor in accelerating progress towards the Social Development Goals (SDG) especially SDGs 1.3, 3.8 and 8.b [41]. Accordingly, public sector investment for improving resilience and social protection policies are urgently needed. These assets are important for building adaptive capacity of individuals and households. Finally, the government needs to consider fiscal measures (e.g., taxes, social transfers) to increase universal social protection and ensuring sustainable domestic financing.

### Conclusion

This study identified a significant association between life satisfaction and various demographic, economic and health-related variables. Changes in employment status and income were found to predict greater challenge in maintaining life satisfaction. This is probably attributable to their limited savings and weak social safety net during the epidemic. Lifestyle changes which led to insufficient physical activity and reduced fruit and vegetable intake had an adverse effect on level of life satisfaction. That change made it more difficult for people to cope with daily life during the epidemic. These findings suggest that policies and strategies which address the root causes of the sociodemographic and socioeconomic disparities and lifestyle changes in the Thai population are needed. Specific recommended actions include building resilience and social protection systems to empower individuals—especially the poor and vulnerable—to cope with a crisis, improve health and wellbeing outcomes, and seek opportunity to lift themselves and their families out of adversity toward a better life.

## Supplementary Material

SUPPLEMENTARY FILE S1 The 2019 Survey questionnaire.
