# Supplementary material for: Life Satisfaction Before and During COVID-19 Pandemic in Thailand
Source: Int J Public Health. 2023 Jul 13;68:1605483. doi: 10.3389/ijph.2023.1605483 (PMC10372220; doi:10.3389/ijph.2023.1605483)
Supplement: Supplementary file 1 [file DataSheet1.PDF]

"Survey Questionnaire on Fruit and Vegetable Consumption among Thai People"

PART 1: GENERAL INFORMATION

1.1: Demographic characteristics

Q1. Sex

- o Male
- o Female

Q2.1 Birth date ___
Q2.2 Birth month ___
Q2.3 Birth year (BE) ___
Q2.4 Age (year) ___

Q3. Marital status

- o Single (including having a partner, but not married)
- o Marriage (both registered and non-registered)
- o Widow
- o Divorce
- o Separated

Q4. Place of residence

- o Urban
- o Rural

1.2: Socio-economic status

Q5. Highest education level

- o Primary school
- o Secondary school
- o Bachelor
- o Higher than bachelor
- o No formal education

Q6. Do you currently have a job?

- o Yes
- o Unemployed / Looking for work
- o Waiting for seasonal job

- No - work at home
- No - study
- No - retired
- No - unable to work due to sickness or disability
- No – too old to work
- Volunteer job
- Others (please specify ___________)

Q7. The average monthly personal income that you earn by yourself. (before deducting monthly expenses) __________ baht

PART 2: HEALTH STATUS

Q8. Do you have chronic disease(s)?

- No
- Yes, please specify all the diseases you have.

PART 3: HEALTH RELATED BEHAVIORS

Q9. Did you do, in the past week, any of exercises, sports, or physically active hobbies (such as brisk walking, running, aerobics, competitive games, sports) at least 30 minutes every day?

- No
- Yes

PART 4: CONSUMPTION OF FRUITS AND VEGETABLES

Q10. Below are questions about vegetable consumption. Please indicate frequency and amount of your vegetable consumption in the past week.

Q10.1 How many days, in the past week, did you eat vegetables?

- Did not eat
- 1 day per week
- 2 days per week
- 3 days per week
- 4 days per week
- 5 days per week
- 6 days per week
- Everyday

Q10.2 How many times a day did you normally eat vegetables? _____ time(s)

Q10.3 How many serving(s) of vegetables did you normally eat each time? _____ serving(s) (number of rice-serving spoons/ladles)

Group	Q10.1 Frequency of vegetable consumption (per week)								Q10.2 Number of times a day to eat	Q10.3 Average amount of vegetable consumption (each time)	
	None	1 day	2 days	3 days	4 days	5 days	6 days	every day		Sample of one rice-serving spoon/ladle*	Number of serving(s) (rice-serving spoon/ladle)
1. Roots (such as carrot, radish, fingerroot, onion, red onion, ginger, galangal)<br>*not including sweet potatoes										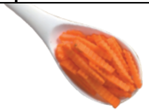<br>1 ladle	
2. Stems and stalks (such as celery, lentils, green/spring onion, garlic chives, morning glory, water mimosa)										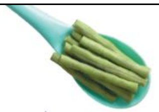<br>1 ladle	
3. Leaves (such as cabbage, kale, spinach, levy gourd, Chinese cabbage (pak-choi, pe-tsai), lettuce, Baegu, Senna siamea, white popinac)										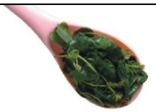<br>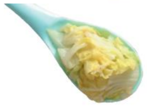<br>1 ladle	
4. Flowers/flower buds with stems/stalks (such as asparagus; broccoli, broccoli raab; Chinese broccoli, cauliflower, green cauliflower, Sesbania grandiflora, Sesbania flower, pumpkin flower, banana blossom, Siamese neem)										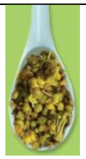<br>1 ladle	

Group	Q10.1 Frequency of vegetable consumption (per week)								Q10.2 Number of times a day to eat	Q10.3 Average amount of vegetable consumption (each time)	
	None	1 day	2 days	3 days	4 days	5 days	6 days	every day		Sample of one rice-serving spoon/ladle*	Number of serving(s) (rice-serving spoon/ladle)
5. Fruits (such as cucumber, zucchini, winter melon, green eggplant, eggplant, bitter melon, pumpkin, tomato, twisted cluster bean, sweet/bell - peppers, green/red/yellow)										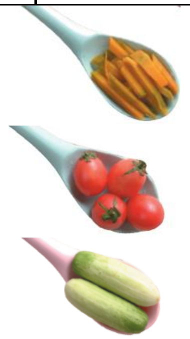 1 ladle	

* Image from Healthy Eating Guidelines (2007) from the Bureau of Nutrition, Department of Health, Ministry of Public Health, Thailand

Q11. Below are questions about fruit consumption. Please indicate frequency and amount of your fruit consumption in the past week.
Q11.1 How many days, in the past week, do you eat fruits?

- Did not eat
- 1 day per week
- 2 days per week
- 3 days per week
- 4 days per week
- 5 days per week
- 6 days per week
- Everyday

Q11.2 How many times a day did you normally eat fruits? _____ time(s)
Q11.3 How many serving(s) of fruits did you normally eat each time? _____ serving(s) Examples of one serving - half a mango, half a Cavendish banana, one cultivated banana, one large tangerine, 6-8 grapes, 4 rambutans, 6 mouth-size pieces of pineapple.
(Based on standard servings in Healthy Eating Guidelines (2007) from the Bureau of Nutrition, Department of Health, Ministry of Public Health, Thailand)

Group	Q11.1 Frequency of fruit consumption (per week)								Q11.2 Number of times a day to eat	Q11.3 Average amount of fruit consumption (each time)	
	None	1 day	2 days	3 days	4 days	5 days	6 days	every day		Sample of one fruit serving*	Number of serving(s) (rice-serving spoon/ladle)
1. Very small-sized fruits (such as grapes, longan, lychee, Jamaican cherry, star gooseberry, Burmese grapes, strawberry, tamarind).										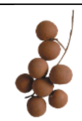 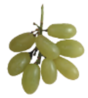	
2. Small-sized fruits (such as rambutan, mangosteen, Marian plum, Sapodilla)										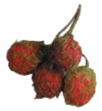 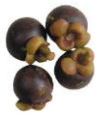	
3. Small-medium-sized fruits (such as cultivated banana, tangerine, apple, pear, star apple)										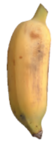 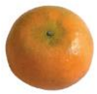	

Group	Q11.1 Frequency of fruit consumption (per week)								Q11.2 Number of times a day to eat	Q11.3 Average amount of fruit consumption (each time)	
	None	1 day	2 days	3 days	4 days	5 days	6 days	every day		Sample of one fruit serving*	Number of serving(s) (rice-serving spoon/ladle)
4. Large-medium-sized fruits (such as Cavendish banana, guava, mango, custard apple)										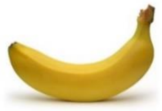 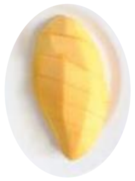	
5. Large-sized fruits (such as watermelon, papaya, pineapple, cantaloupe, coconut)										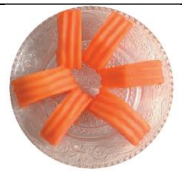 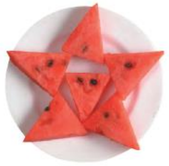	

* Image from Healthy Eating Guidelines (2007) from the Bureau of Nutrition, Department of Health, Ministry of Public Health, Thailand

PART 5: The Satisfaction with Life Scale

In the past month. How do you agree with this text?

Q12. In most ways my life is close to my ideal.

- ○ 1 - Strongly disagree
- ○ 2 – Disagree
- ○ 3 - Slightly disagree
- ○ 4 - Neither agree nor disagree
- ○ 5 - Slightly agree
- ○ 6 – Agree
- ○ 7 - Strongly agree

Q13. The conditions of my life are excellent.

- ○ 1 - Strongly disagree
- ○ 2 – Disagree
- ○ 3 - Slightly disagree
- ○ 4 - Neither agree nor disagree
- ○ 5 - Slightly agree
- ○ 6 – Agree
- ○ 7 - Strongly agree

Q14. I am satisfied with my life.

- ○ 1 - Strongly disagree
- ○ 2 – Disagree
- ○ 3 - Slightly disagree
- ○ 4 - Neither agree nor disagree
- ○ 5 - Slightly agree
- ○ 6 – Agree
- ○ 7 - Strongly agree

Q15. So far I have gotten the important things I want in life.

- 1 - Strongly disagree
- 2 – Disagree
- 3 - Slightly disagree
- 4 - Neither agree nor disagree
- 5 - Slightly agree
- 6 – Agree
- 7 - Strongly agree

Q16. If I could live my life over, I would change almost nothing.

- 1 - Strongly disagree
- 2 – Disagree
- 3 - Slightly disagree
- 4 - Neither agree nor disagree
- 5 - Slightly agree
- 6 – Agree
- 7 - Strongly agree
